# Specific absorbed fractions and radionuclide S values for adult and pediatric respiratory tracts within ICRP series of mesh-type reference computational phantoms

**DOI:** 10.1088/1361-6498/ae1843

**Published:** 2025-11-06

**Authors:** Bangho Shin, Chansoo Choi, Robert J Dawson, Chan Hyeong Kim, Wesley E Bolch

**Affiliations:** 1 J. Crayton Pruitt Family Department of Biomedical Engineering, University of Florida, Gainesville, FL, United States of America; 2 Medical Physics Program College of Medicine, University of Florida, Gainesville, FL, United States of America; 3 Department of Nuclear Engineering, Hanyang University, Seoul, Republic of Korea

**Keywords:** internal dosimetry, respiratory tract, specific absorbed fraction, *S* value, ICRP mesh phantom

## Abstract

Recently, the International Commission on Radiological Protection (ICRP) has released adult and pediatric mesh-type reference computational phantoms (MRCPs) through its Publications 145 and 156, which incorporate anatomically refined respiratory tract structures that overcome the limitations of earlier voxel and stylized models. In this study, a comprehensive dataset of specific absorbed fractions (SAFs) and radionuclide *S* values was generated for the respiratory tract across the entire age- and sex-specific series of ICRP MRCPs. The phantoms were implemented in the Geant4 Monte Carlo radiation transport code (version 11.3) to compute SAFs for photons, electrons, and alpha particles over the energy ranges of 0.001–10 MeV for photons and electrons and 1–12 MeV for alpha particles, with certain low-energy values supplemented by a limiting SAF interpolation approach. The calculated SAFs were subsequently combined with nuclear decay data from ICRP Publication 107 to derive S values for all relevant source regions following inhalation exposures to radionuclides. Photon and electron SAFs were obtained for 36 source–target combinations, and alpha SAFs for 18 combinations, while *S* values were produced for 1,252 radionuclides. The calculated SAFs exhibited clear age-dependent trends, with larger values in younger phantoms. Furthermore, the calculated SAFs and *S* values were generally greater than previously reported ICRP values. The complete dataset is available through an open-access repository, representing the first effort to provide SAFs and *S* values for the respiratory tract using the ICRP MRCPs. The calculations explicitly accounted for micrometre-scale source and target regions within anatomically realistic respiratory tract structures, while also incorporating inter-tissue irradiation cases, which had not been possible with previous models.

## 1. Introduction

The respiratory tract is the most critical and frequent pathway for radionuclide intake into the human body, posing a risk of internal exposure to both workers and the general public across nuclear and radiation industries, medical imaging and therapy, environmental contamination, and radiological emergencies (Toohey et al 1991, ICRP 1994, Patni et al 2013). The calculation of dose coefficients from internally incorporated radionuclides requires information on their time-dependent biokinetic behaviour within the body (ICRP 2015), physical decay properties (ICRP 2008), and the fraction of emitted energy deposited per unit mass of the target region (ICRP 2016a, 2023). The final attribute, known as the specific absorbed fraction (SAF), is derived using computational models that serve as human anatomical surrogates, in combination with Monte Carlo radiation transport simulations. Publications 133 and 155 of the International Commission on Radiological Protection (ICRP) (ICRP 2016a, 2023) provide an extensive dataset of SAFs for ICRP reference individuals (ICRP 2002), including adults and children, calculated primarily using the voxel-type reference computational phantoms (VRCPs) described in ICRP Publications 110 and 143, respectively (ICRP 2009, 2020a). The ICRP-133 SAFs (ICRP 2016a) have been utilized, in combination with biokinetic models (ICRP 2015) and physical decay data (ICRP 2008), to derive dose coefficients in the occupational intakes of radionuclides series (ICRP 2015, 2016b, 2017, 2019, 2022). In addition, the ICRP-155 SAFs (ICRP 2023) are intended to support the production of dose coefficients in the forthcoming environmental intakes of radionuclides series.

The VRCPs (ICRP 2009, 2020a), primarily used in these SAF calculations (ICRP 2016a, 2023), were developed from computed tomography (CT) images of real individuals, offering improved anatomical realism over earlier stylized phantoms. However, they do not faithfully represent organs and tissues with fine structures, particularly those smaller than their voxel resolution (e.g. adult female: 1.775 × 1.775 × 4.84 mm^3^). For example, micrometre-thick source and radiosensitive target regions in the respiratory and alimentary tracts (ICRP 1994, 2006) cannot be represented within the structure of the whole-body phantoms, which prevents adequate dosimetry for weakly penetrating radiations such as electrons and alpha particles that require explicit consideration of these regions. Therefore, for electrons and alpha particles in the respiratory and alimentary tracts, SAFs were calculated using the stylized models provided in ICRP Publications 66 and 100 (ICRP 1994, 2006), respectively. These stylized models represent the organs and tissues within these tracts using simplified geometries such as spheres and right cylinders. For example, the bronchial (BB) region is represented by a stylized model consisting of ten concentric cylindrical layers, encompassing both the target and source regions. While these stylized models support dosimetry that considers thin regions for weakly penetrating radiations, they lack anatomical realism and cannot account for cross-fire irradiation not only between surrounding anatomical structures but also within the same structure (e.g. the highly branched BB and bronchiolar (bb) regions).

To address these limitations, the present study updates SAFs for the respiratory tract using the mesh-type reference computational phantoms (MRCPs) recently introduced in ICRP Publications 145 and 156 (ICRP 2020b, 2024). The MRCPs were developed to overcome the anatomical and dosimetric limitations of the VRCPs, employing the tetrahedral-mesh (TM) format, which is regarded as the most advanced modelling format in computational dosimetry (Kainz et al 2019). One of the key advantages of the MRCPs lies in their capability to represent fine anatomical structures, even micrometre-thick regions within the respiratory and alimentary tracts (Kim et al 2017, Choi et al 2022, 2023), thereby supporting dosimetry that explicitly considers thin regions while maintaining anatomical fidelity. This capability of the MRCPs has also been utilized in recent efforts to update SAFs for the alimentary tract (Kwon et al 2024). In the present study, the SAFs for the respiratory tract were calculated using the Geant4 Monte Carlo radiation transport code (Allison et al 2016), for monoenergetic photons and electrons (0.001–10 MeV) and alpha particles (1–12 MeV). These SAFs were then combined with the physical decay data of 1,252 radionuclides (ICRP 2008) to derive corresponding *S* values. Finally, the resulting SAFs and *S* values were compared with previously published ICRP data (ICRP 2016a, 2023) to assess their dosimetric implications.

## 2. Materials and Methods

### 2.1. Respiratory tract of MRCPs

**Figure 1.**
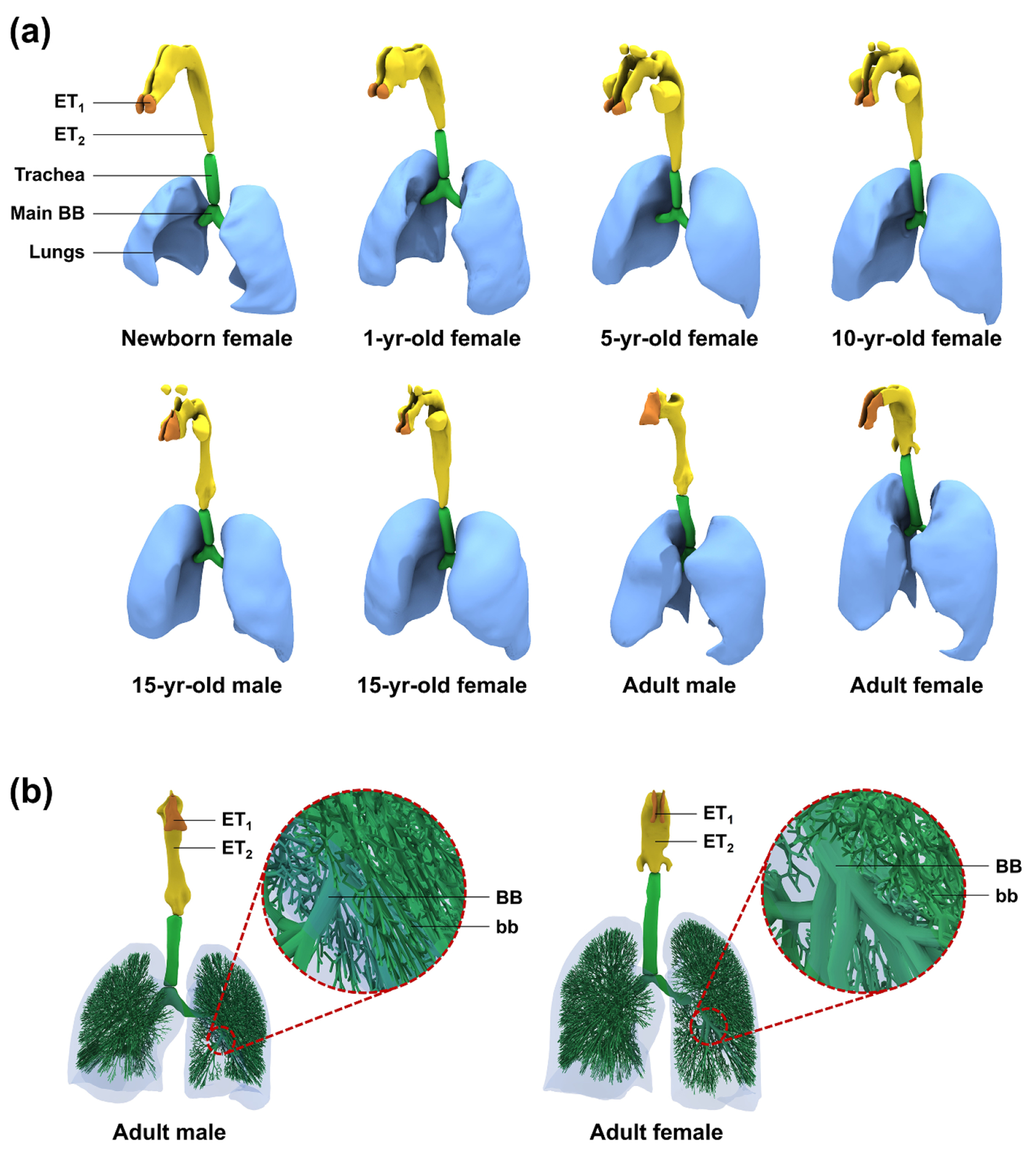
(a) Overview of respiratory tract in mesh-type reference computational phantoms (MRCPs), and (b) lung airways from generation 2–15 within the lungs, shown here for the adult male and female only. In this figure, constructive solid geometry (CSG) format of lung airways was converted into polygonal mesh format for visualization purpose.

Figure 1(a) shows the anatomical structures of the respiratory tract in the adult and pediatric MRCPs of ICRP Publications 145 and 156 (ICRP 2020b, 2024)—representing both sexes for newborn (00 M/F), 1 year (01 M/F), 5 years (05 M/F), 10 years (10 M/F), 15 years (15 M/F), and adult (AM/AF)—including the extrathoracic regions (ET_1_ and ET_2_), trachea (generation 0), main BB region (generation 1), and the outer contours of the left and right lungs. These structures were directly converted into high-quality mesh representations from their counterparts in the VRCPs of ICRP Publications 110 and 143 (ICRP 2009, 2020a), with the exception of the pediatric ET_2_, which was substantially refined based on updated anatomical data (Choi et al 2023). In addition, as shown in figure 1(b), the MRCPs explicitly define the remaining BB region (generation from 2 to 8) and bb region (generation from 9 to 15) within the lungs, which are not represented in the VRCPs. While all other anatomical structures in the MRCPs are modelled using the TM format, these lung airways are uniquely represented using the constructive solid geometry (CSG) format. In this format, primitive 3D solids are volumetrically combined by performing one or more Boolean operations. In the case of the MRCP airways, the CSG model was generated by performing serial Boolean union operations on the individual airway segments defined in ICRP Publications 145 and 156 (ICRP 2020b, 2024). This modelling approach is uniquely possible for the airways given that the primitive geometric elements are truncated cones with prescribed start points, end points, and radii for the top and bottom faces. This modelling choice was made to reduce computer memory requirements and, ultimately, to support more efficient Monte Carlo radiation transport simulations (Kim et al 2017, Choi et al 2023). The rest of the lungs other than the airways is defined as alveolar-interstitial (AI) region.

**Figure 2.**
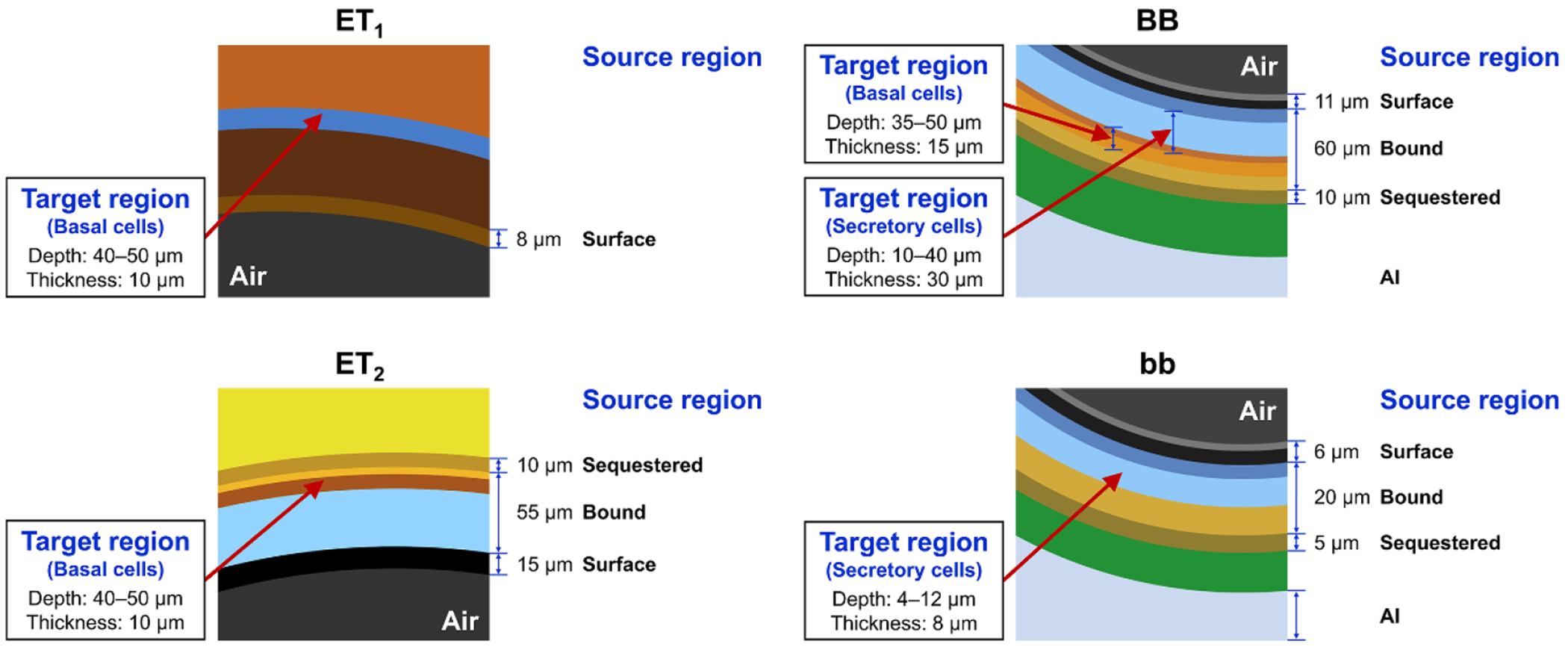
Illustration of micrometre-thick source and target regions in respiratory tract of mesh-type reference computational phantoms (MRCPs).

Figure 2 illustrates the micrometre-thick source and target regions defined within the ET_1_, ET_2_, BB, and bb regions of the MRCPs. The depths of these regions were directly adopted from ICRP Publication 66 (ICRP 1994) and are identical across all ages. Table 1 summarizes the masses of the target regions for each organ, together with the corresponding values reported in ICRP Publications 133 and 155 (ICRP 2016a, 2023). For the adult MRCPs, the BB and bb regions were first defined by Kim et al (2017) but were slightly revised prior to their official release in ICRP Publication 145 (2020b). As part of the ICRP’s effort to define all organs as blood-inclusive, the morphologies of many organs including the lungs underwent minor adjustments. Accordingly, the airways were regenerated using the same dimensions adopted by Kim et al (2017), and the finalized model values are those reported in this table. The values for the ET_1_ and ET_2_ regions of the adult MRCPs, as well as for all regions of the pediatric MRCPs, are adopted directly from those reported by Kim et al (2017) and Choi et al (2023), respectively. As shown in this table, the MRCP values are generally much smaller than the ICRP-133/155 values. Note that, except for the newborn, the ICRP-133/155 values were inherited directly from the ICRP-66 values, which had been derived by scaling down the values of the adult male stylized models, as stylized models were available only for the adult male. Especially, the adult ET_2_ region exhibits the most pronounced difference, with values differing by ~5 times. This substantial difference arises from morphological discrepancies between the MRCPs and the stylized models, as the former were constructed from CT images of real individuals, whereas the latter were represented by simplified geometries.

### 2.2. Production of SAFs and S value dataset

The present study established photon, electron, and alpha SAFs, which can be expressed as the fraction of energy emitted from a source region that is absorbed per unit mass of a target region, for the respiratory tract using the adult and pediatric MRCPs (ICRP 2020b, 2024). Then, using the established SAFs, the *S* value, which is defined as the mean absorbed dose in a target region per decay in a source region, was produced for 1,252 radionuclides provided in ICRP Publication 107 (ICRP 2008).

**Figure 3.**
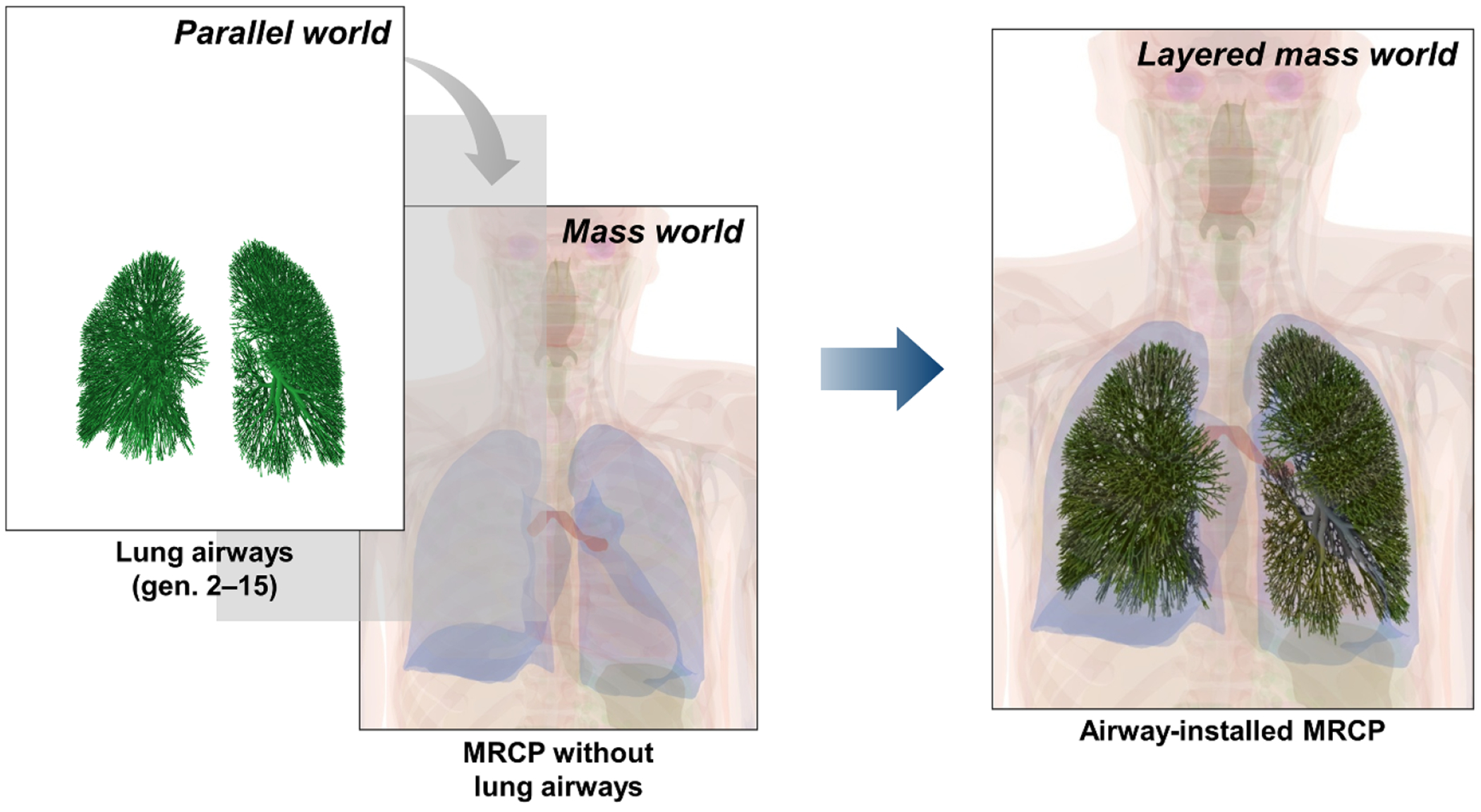
Installation process of lung airways (generation from 2 to 15) into lungs of mesh-type reference computational phantoms (MRCPs) using parallel world feature of Geant4 Monte Carlo code.

For SAF calculations, the present study employed the Geant4 Monte Carlo code (version 11.3, released in December 2024) (Allison et al 2016). The Geant4 code provides a parallel geometry feature, which allows the distinct geometries to coexist by overlaying the geometry in the parallel world onto that in the mass world (Apostolakis et al 2008). The CSG-based airways were implemented in the parallel world using *G4Cons, G4Sphere*, and *G4BooleanSolid* classes, while the TM-based MRCPs were implemented in the mass world using *G4Tet* and *G4PVParameterisation* classes (see figure 3). The phantoms were assumed to be placed in the vacuum environment as the surrounding medium.

After the phantom implementation, the mono-energetic source particles (0.01–10 MeV for photons and electrons and 2–12 MeV for alpha particles) were generated and emitted isotropically using *G4VUserPrimaryGeneratorAction* class. The source particles were uniformly sampled in the source regions using the barycentric coordinate system-based sampling method (Rocchini and Cignoni 2000). The particles were transported using the *QGSP_BIC_LIV* physics list provided in the Geant4 code. The secondary range cut values were set to 0.1 *μ*m for photons, electrons, positrons, and protons. To calculate the SAFs, energy depositions in the target regions were scored using the *G4PSEnergyDeposit* class. 10^4^–10^9^ source particles were transported to keep the statistical relative errors of the calculated SAFs below 5%. The simulations were performed on four desktop computers, two equipped with an Intel^®^ Core^™^ i7-14700K processor (8 performance cores, 12 efficient cores, 28 threads, base frequency of 3.4 GHz for performance core, base frequency of 2.5 GHz for efficient core, max turbo frequency of 5.6 GHz) and 64 GB of RAM and two equipped with an Intel^®^ Core^™^ i7-12700 processor (8 performance cores, 4 efficient cores, 20 threads, base frequency of 2.1 GHz for performance core, base frequency of 1.6 GHz for efficient core, max turbo frequency of 4.9 GHz) and 32 GB of RAM. Variance reduction techniques were not used in Monte Carlo simulations.

For low-energy ranges, the statistical relative errors of some SAFs exceeded 5% even with 10^9^ source particles, resulting in some data points being unavailable in the dataset. To establish a complete SAF dataset (0.001–10 MeV for photons and electrons and 1–12 MeV for alpha particles), the present study adopted a limiting SAF for low-energy interpolation, following the methods adopted by the ICRP (ICRP 2016a, Schwarz et al 2021, Kwon et al 2024). For cross-fire irradiation cases, where the source and target regions are distinct, the limiting SAF was set to 10^−12^ kg^−1^ at 1 eV. For self-irradiation cases, where the target region lies within the source region, the limiting SAF was set to the reciprocal of the mass of the source region. The missing SAFs were estimated using log–log interpolation between the limiting SAF and the lowest-energy SAF calculated directly from the Geant4 simulations.

After establishing the full SAF dataset, the radionuclide *S* values were produced using the following equation:

$$S\left(r_{\text{T}}\leftarrow r_{\text{S}}\right)=\underset{i}{\sum }E_{\text{R},i}Y_{\text{R},i}\Phi \left(r_{\text{T}}\leftarrow r_{\text{S}},E_{R,i}\right)$$

where $S\left(r_{\text{T}}\leftarrow r_{\text{S}}\right)$ is the S value, *r*_T_ is the target region, *r*_S_ is the source region, *E*_R,i_ is the energy of *i*th radiation of type *R* emitted, *Y_R,i_* is the yield of *i*th radiation of type *R*, and $\Phi \left(r_{\text{T}}\leftarrow r_{\text{S}},E_{R,i}\right)$ is the SAF. An in-house Python script was developed to derive S values using the energy spectrum and yields of each radionuclide specified in ICRP Publication 107 (ICRP 2008) and the established SAFs. Piecewise cubic Hermite interpolating polynomial interpolation was employed to derive the energy points specified in the ICRP-107 energy spectrum.

## 3. Results and discussion

### 3.1. Photon, electron, and alpha SAFs and radionuclide S value dataset

In the present study, we produced a comprehensive dataset of photon, electron, and alpha SAFs (unit: kg^−1^) for the respiratory tract using the adult and pediatric MRCPs (ICRP 2020b, 2024) in combination with the Geant4 Monte Carlo code (Allison et al 2016). The photon and electron SAFs were calculated for 36 source-target combinations across 27 energy points ranging from 0.001 to 10 MeV, while the alpha SAFs were calculated for 18 source-target combinations across 23 energy points ranging from 1 to 12 MeV. At low-energy ranges, particles often fail to interact within very thin tissue layers, resulting in high statistical relative errors even with a very large number of source particles. In such cases, the SAFs with statistical relative errors exceeding 5% were derived using the limiting SAF interpolation methods (ICRP 2016a, Schwarz et al 2021, Kwon et al 2024).

A notable advance of the present dataset is that it provides SAFs for inter-tissue irradiation cases (e.g. BB secretory cells from bb surface) as well as for intra-tissue irradiation cases (e.g. BB secretory cells from BB surface). Such data were unavailable to be directly calculated from the Monte Carlo simulations in the previous ICRP-133/155 SAFs (ICRP 2016a, 2023) due to the adoption of tissue-specific stylized models provided in ICRP Publication 66 (ICRP 1994). To our knowledge, this is the first effort to directly compute SAFs for the inter-tissue irradiation cases for the respiratory tract using whole-body phantoms.

In addition to the SAF dataset, the present study produced *S* values (unit: mGy·MBq^−1^·s^−1^) for 1,252 radionuclides by combining the SAFs with the physical decay data given in ICRP Publication 107 (ICRP 2008). The complete SAFs and *S* value dataset are provided via a GitHub repository available online (https://github.com/BH-Shin/JRP-respiratory_tract_SAFs_Svalues.git). Note that although the dataset includes the interpolated SAFs, all comparisons in this paper were made only for the SAFs from the direct Geant4 simulations for systematic analysis.

### 3.2. Age-dependent trends in SAFs

**Figure 4.**
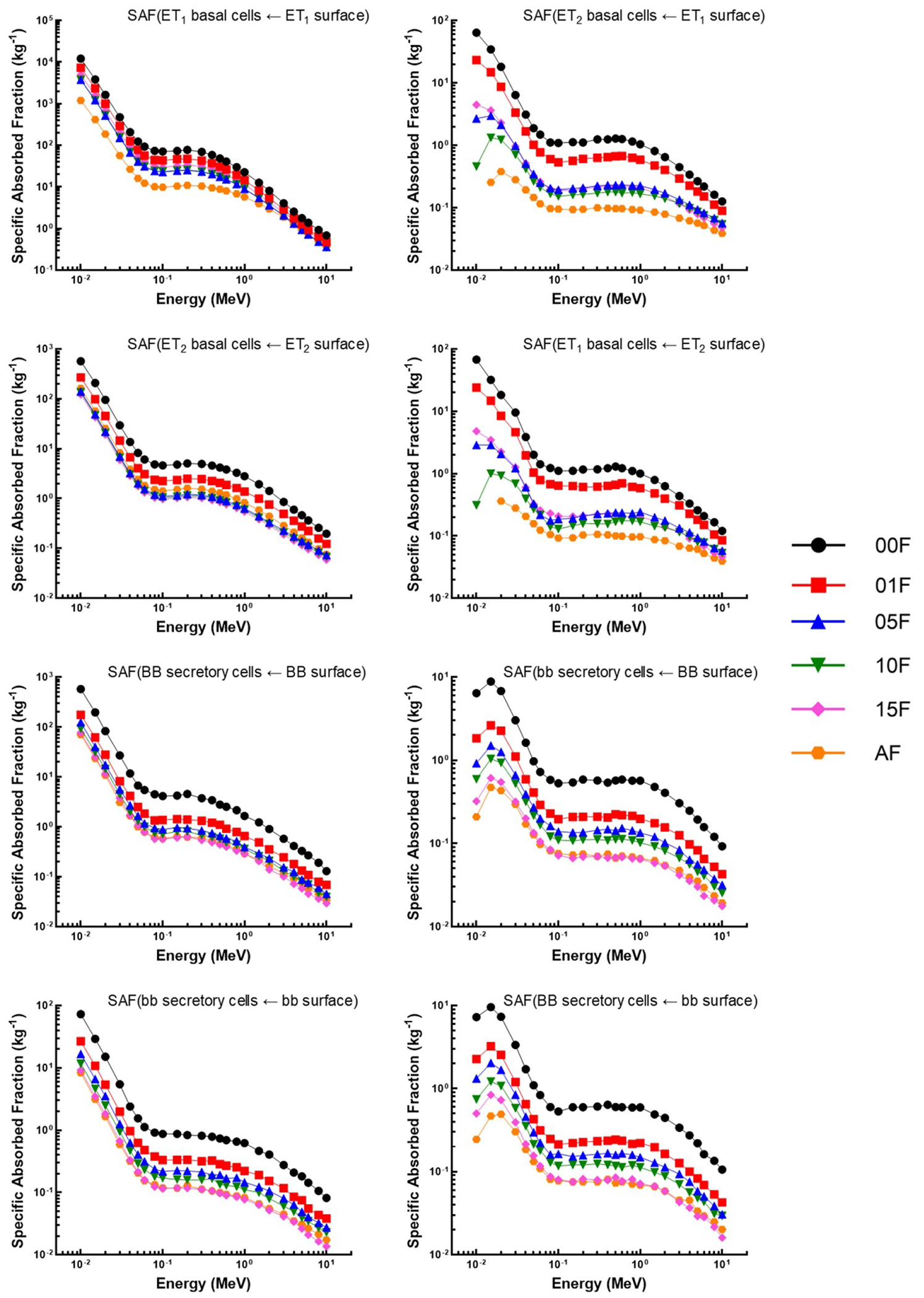
Photon specific absorbed fractions (SAFs) of mesh-type reference computational phantoms (MRCPs) across all female age groups.

**Figure 5.**
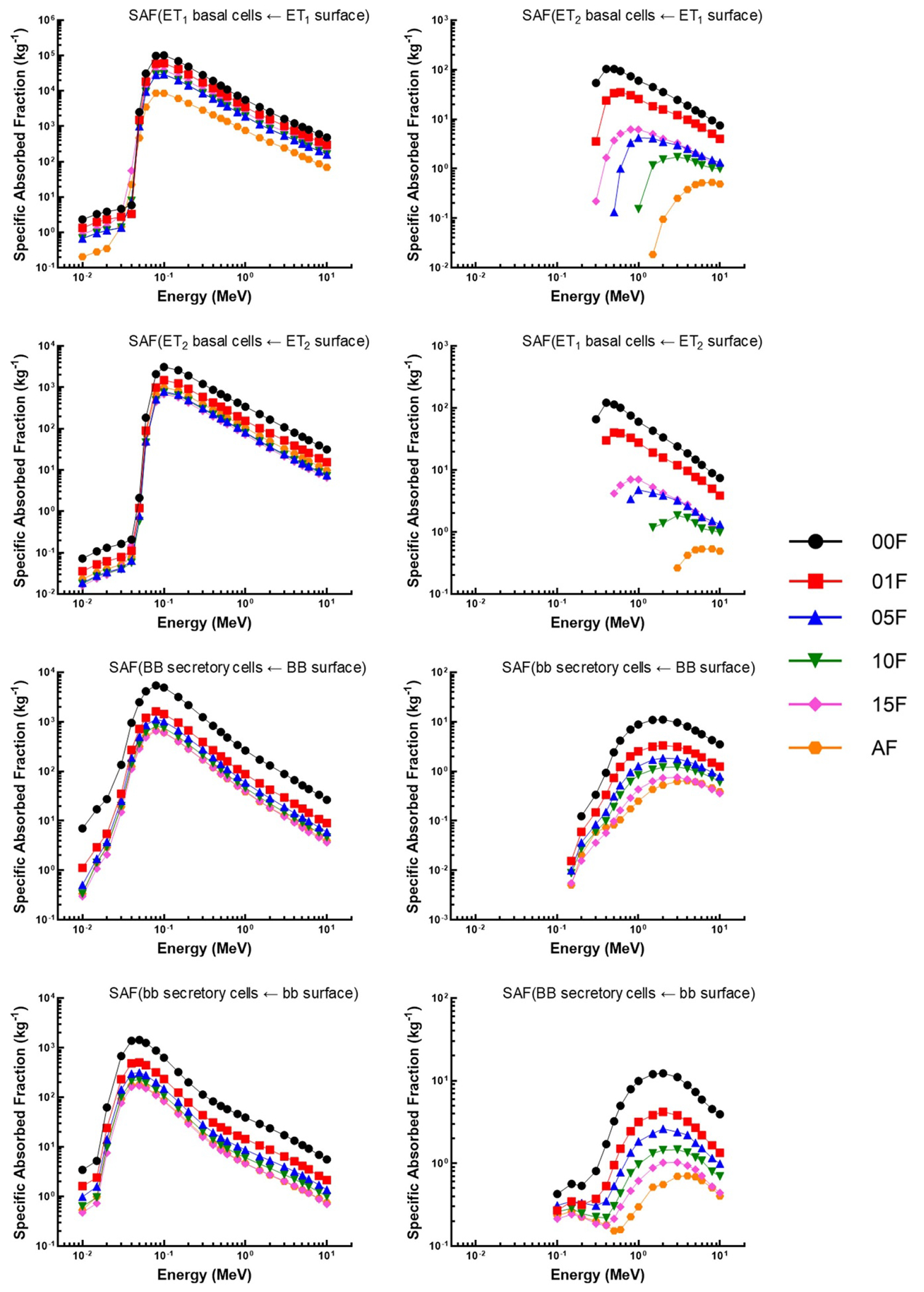
Electron specific absorbed fractions (SAFs) of mesh-type reference computational phantoms (MRCPs) across all female age groups.

**Figure 6.**
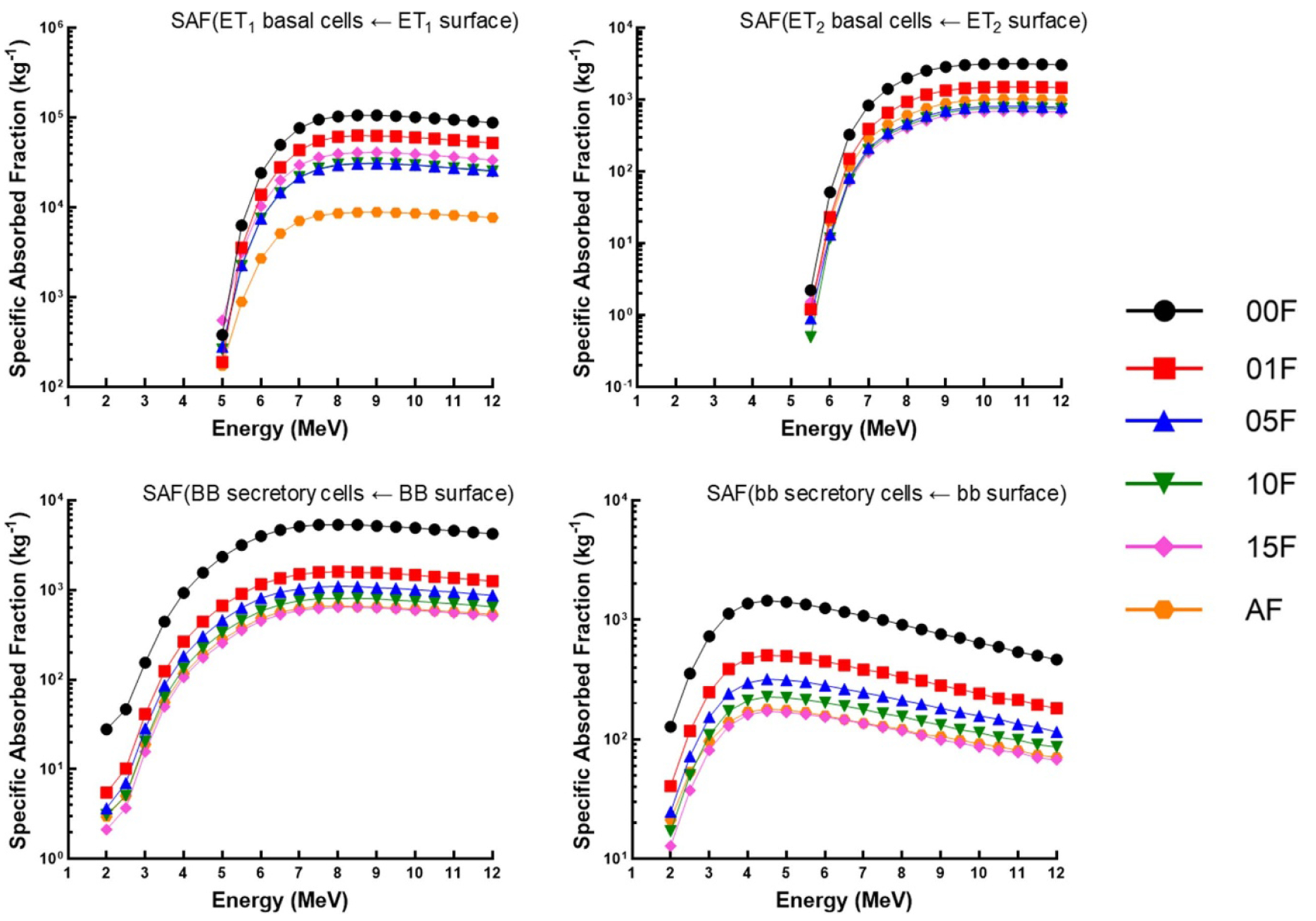
Alpha specific absorbed fractions (SAFs) of mesh-type reference computational phantoms (MRCPs) across all female age groups.

Figures 4–6 present the SAFs for photons, electrons, and alpha particles, respectively, across all female age groups, serving as representative examples for the investigation of age-dependent trends. In these figures, the surface region was selected as the source region for all tissues, while the basal cells for the ET_1_ and ET_2_ regions and the secretory cells for the BB and bb regions were selected as the target regions. The graphs in the left column of each figure show intra-tissue irradiation cases, whereas the graphs in the right column show inter-tissue irradiation cases, except for alpha particles, for which inter-tissue irradiation was negligible and thus only intra-tissue irradiation cases are shown.

Overall, these figures show general trends of increasing SAFs with decreasing age across all particles and energies. In intra-tissue irradiation cases, the differences are driven mainly by the smaller target masses at younger ages (see table 1), despite relatively modest variations in absorbed fractions (AFs). For inter-tissue irradiation, the age-dependent differences are even more pronounced, reflecting not only the influence of smaller target masses but also the closer spatial proximity of tissues at younger ages, which leads to larger AFs. For the ET_1_ and ET_2_ regions, however, the age dependency becomes less evident, with reversals also observed, beyond 5 years of age. This observation can be explained by the fact that the dimensions of the head, where ET regions are primarily located, only gradually increase after 5 years (Roche et al 1987), which may have introduced some influence from individual anatomical variability in the CT images used for the development of the MRCPs. Nevertheless, as the tissue weighting factor for the entire ET region is below 0.01, the impact of such variability on effective dose calculations is likely to be minor. Meanwhile, the BB and bb regions, with relatively dominant tissue weighting factors (0.12 for the lungs), display clearer age-dependent trends, except for the 15 year-old female, whose body dimensions are nearly identical to those of the adult female, resulting in similar or in some cases reversed patterns.

### 3.3. Comparison of absorbed fractions and SAFs with ICRP Publications 133 and 155

**Figure 7.**
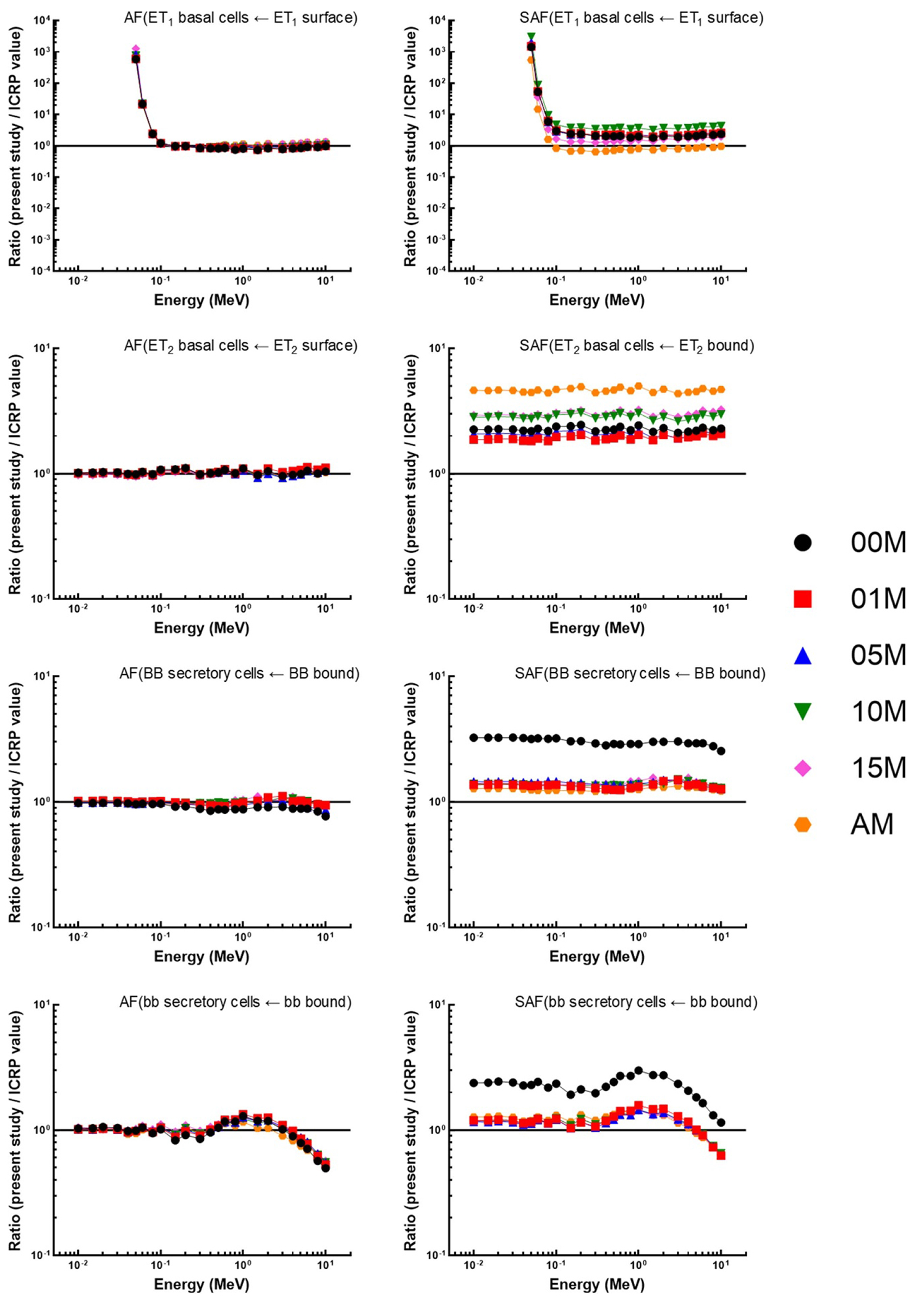
Ratio of electron absorbed fractions (AFs) and specific absorbed fractions (SAFs) of mesh-type reference computational phantoms (MRCPs) to those of ICRP-133/155 values across all male age groups.

**Figure 8.**
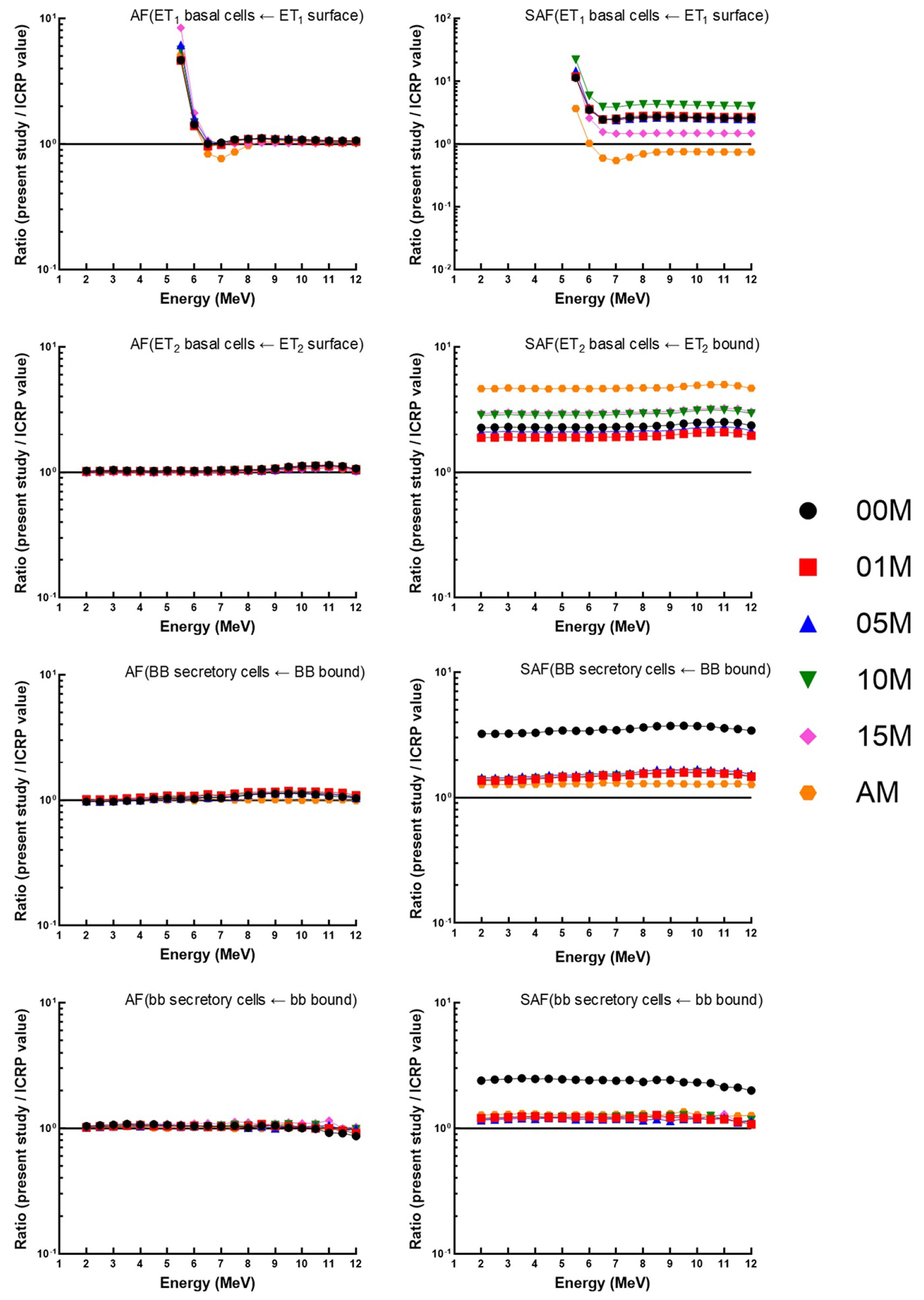
Ratio of alpha absorbed fractions (AFs) and specific absorbed fractions (SAFs) of mesh-type reference computational phantoms (MRCPs) to those of ICRP-133/155 values across all male age groups.

Figures 7 and 8 show the ratios of the AFs and the SAFs calculated in the present study to those reported in ICRP Publications 133 and 155 (ICRP 2016a, 2023) for electrons and alpha particles, respectively, across all male age groups. In these figures, the bound region was selected as the source region for all tissues, except for ET_1_ region, for which the surface region is the only source region considered. The basal cells for the ET_1_ and ET_2_ regions and the secretory cells for the BB and bb regions were selected as the target regions. The graphs in the left column of each figure show the AF ratios, whereas the graphs in the right column show SAF ratios. Note that the ICRP-133/155 adult SAFs have been confirmed to be identical.

The AF figures show that most of the ratios are close to unity, despite the differences in the models between the MRCPs and the stylized models. This is mainly attributed to the utilization of the same depth and thickness for the source and target regions. At higher energies, where particles emitted from the source region can travel longer distances, the AF differences for the airway regions are influenced by anatomical geometry, particularly in the bb region; the MRCPs capture a highly branched structure confined within the actual lung volume, whereas the stylized models approximate it as a cylindrical region embedded within an infinite lung volume. In the ET_1_ region, where the target region is separated from the source region by tens of micrometres unlike in other cases, the pronounced AF differences observed at low electron (<100 keV) and alpha particle (<6 MeV) energies are most likely attributable to differences in the physics models employed, a finding consistent with previous reports (Kim et al 2017, Choi et al 2022, 2023, Kwon et al 2024). Note that the ICRP-133/155 calculations were performed with the MCNPX code (version 2.6, released in April 2008) (Pelowitz 2008). Previous work validated that such significant differences can arise solely from the physics model used, even when identical stylized models are applied, although this validation was limited to electrons (Choi et al 2022, 2023).

The SAF figures further highlight the influence of the target masses. These figures show that most of the ratios exceed unity, indicating that the SAFs calculated in the present study are generally larger than the ICRP-133/155 values, as the MRCP-based target masses are generally much smaller than those of the stylized models (see table 1). An exception is observed for the ET_1_ region of the adult male, in which the larger target mass leads to ratios falling below unity.

### 3.4. Comparison of S values with ICRP Publications 133 and 155

**Figure 9.**
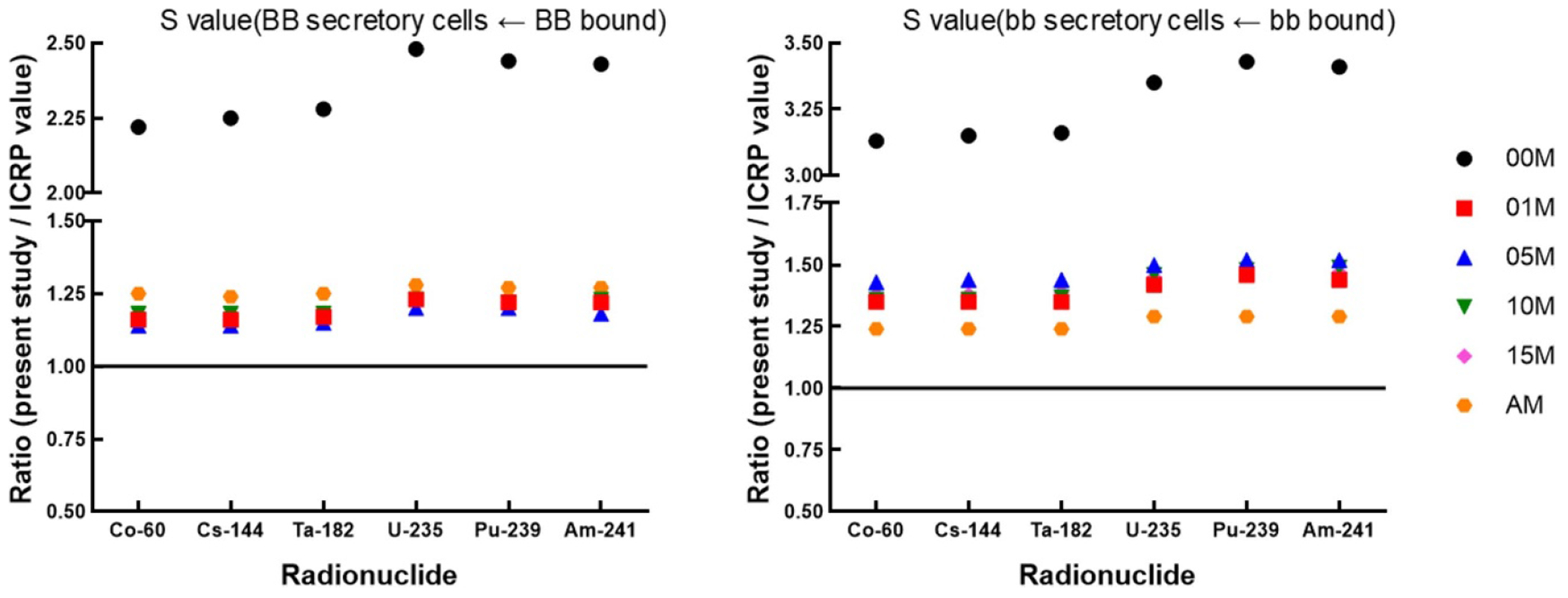
Ratio of *S* values of mesh-type reference computational phantoms (MRCPs) to those calculated from ICRP-133/155 values for ^60^Co, ^144^Ce, ^182^ Ta, ^235^U, ^239^Pu, and ^241^Am across all male age groups.

Figure 9 shows the ratios of the S values for ^60^Co, ^144^ Ce, ^182^Ta, ^235^ U, ^239^Pu, and ^241^Am calculated in the present study to those calculated using the ICRP-133/155 SAFs across all male age groups. Considering that the selected radionuclides are known to be retained in the thoracic airways via inhalation (ICRP 2015), the source and target regions were selected to bound region and secretory cells for the BB and bb regions, respectively. The intra-tissue irradiation cases were considered for comparison. As expected, the figure shows that the S value ratios are consistently greater than unity for all cases, with the newborn male showing the highest values. These ratios are in line with the results observed for the SAFs, reflecting primarily the differences in the target mass and the anatomical geometry (cylinder vs. tree-like branches).

## 4. Conclusion

In the present study, we produced a comprehensive set of photon, electron, and alpha SAFs and radionuclide *S* values for the respiratory tract using the adult and pediatric MRCPs and the Geant4 Monte Carlo code. A novel feature of the produced dataset is the inclusion of inter-tissue irradiation cases, in addition to intra-tissue irradiation cases, calculated using whole-body phantoms for the first time. The SAFs generally revealed age-dependent trends, with younger ages exhibiting larger SAFs due to smaller target masses and closer tissue proximities. Comparisons with the ICRP-133/155 values demonstrated that the present SAFs and S values generally showed higher values, reflecting the differences in the target masses and the anatomical geometry, particularly in the BB and bb regions where the MRCPs represent the tree-like branching airway structures. The authors believe that the produced dataset will be beneficially used to estimate doses for the respiratory tract for internal exposures, such as inhaled radionuclides and radiopharmaceuticals. In addition, the dataset can serve as a valuable reference for future ICRP dose coefficient calculations and radiological protection applications.

## Figures and Tables

**Table 1.** Masses (unit: gram) of target regions in respiratory tract of adult and pediatric mesh-type reference computational phantoms (MRCPs), together with corresponding values reported in ICRP Publications 133 and 155 (ICRP 2016a, 2023).

Region	Target	00M		01M		05M		10M		15M		AM	
		MRCP	Stylized	MRCP	Stylized	MRCP	Stylized	MRCP	Stylized	MRCP	Stylized	MRCP	Stylized
ET_1_	Basal cells	9.8 × 10^−4^	2.4 × 10^−3^	1.6 × 10^−3^	4.1 × 10^−3^	3.5 × 10^−3^	8.3 × 10^−3^	3.3 × 10^−3^	1.3 × 10^−2^	1.3 × 10^−2^	1.9 × 10^−2^	2.8 × 10^−2^	2.0 × 10^−2^
ET_2_	Basal cells	2.4 × 10^−2^	5.3 × 10^−2^	5.0 × 10^−2^	9.3 × 10^−2^	9.2 × 10^−2^	1.9 × 10^−1^	1.0 × 10^−1^	2.8 × 10^−1^	1.4 × 10^−1^	4.2 × 10^−1^	9.8 × 10^−2^	4.5 × 10^−1^
BB	Basal cells	3.5 × 10^−2^	1.1 × 10^−1^	1.1 × 10^−1^	1.6 × 10^−1^	1.6 × 10^−1^	2.3 × 10^−1^	2.3 × 10^−1^	3.1 × 10^−1^	2.9 × 10^−1^	4.1 × 10^−1^	3.4 × 10^−1^	4.3 × 10^−1^
	Secretory cells	6.7 × 10^−2^	2.2 × 10^−1^	2.3 × 10^−1^	3.1 × 10^−1^	3.2 × 10^−1^	4.7 × 10^−1^	4.5 × 10^−1^	6.2 × 10^−1^	5.8 × 10^−1^	8.2 × 10^−1^	6.7 × 10^−1^	8.6 × 10^−1^
bb	Secretory cells	1.7 × 10^−1^	3.9 × 10^−1^	5.1 × 10^−1^	6.0 × 10^−1^	8.3 × 10^−1^	9.5 × 10^−1^	1.1 × 10^0^	1.3 × 10^0^	1.6 × 10^0^	1.8 × 10^0^	1.5 × 10^0^	1.9 × 10^0^
AI^a^	AI^a^	5.8 × 10^1^	5.2 × 10^1^	1.4 × 10^2^	1.5 × 10^2^	2.9 × 10^2^	3.0 × 10^2^	4.8 × 10^2^	5.0 × 10^2^	8.8 × 10^2^	8.6 × 10^2^	1.2 × 10^3^	1.1 × 10^3^
		00F		01F		05F		10F		15F		AF	
Region	Target	MRCP	Stylized	MRCP	Stylized	MRCP	Stylized	MRCP	Stylized	MRCP	Stylized	MRCP	Stylized

ET_1_	Basal cells	9.7 × 10^−4^	2.4 × 10^−3^	1.6 × 10^−3^	4.1 × 10^−3^	3.3 × 10^−3^	8.3 × 10^−3^	3.3 × 10^−3^	1.3 × 10^−2^	2.5 × 10^−3^	1.7 × 10^−2^	1.1 × 10^−2^	1.7 × 10^−2^
ET_2_	Basal cells	2.4 × 10^−2^	5.3 × 10^−2^	5.0 × 10^−2^	9.3 × 10^−2^	9.3 × 10^−2^	1.9 × 10^−1^	9.7 × 10^−2^	2.8 × 10^−1^	1.1 × 10^−1^	3.8 × 10^−1^	7.2 × 10^−2^	3.9 × 10^−1^
BB	Basal cells	3.4 × 10^−2^	1.1 × 10^−1^	1.2 × 10^−1^	1.6 × 10^−1^	1.7 × 10^−1^	2.3 × 10^−1^	2.2 × 10^−1^	3.1 × 10^−1^	2.8 × 10^−1^	3.8 × 10^−1^	2.7 × 10^−1^	3.9 × 10^−1^
	Secretory cells	6.7 × 10^−2^	2.2 × 10^−1^	2.3 × 10^−1^	3.1 × 10^−1^	3.3 × 10^−1^	4.7 × 10^−1^	4.4 × 10^−1^	6.2 × 10^−1^	5.6 × 10^−1^	7.6 × 10^−1^	5.4 × 10^−1^	7.8 × 10^−1^
bb	Secretory cells	1.8 × 10^−1^	3.9 × 10^−1^	5.0 × 10^−1^	6.0 × 10^−1^	7.9 × 10^−1^	9.5 × 10^−1^	1.1 × 10^0^	1.3 × 10^0^	1.5 × 10^0^	1.6 × 10^0^	1.4 × 10^0^	1.9 × 10^0^
AI^a^	AI^a^	5.8 × 10^1^	5.2 × 10^1^	1.4 × 10^2^	1.5 × 10^2^	2.9 × 10^2^	3.0 × 10^2^	4.9 × 10^2^	5.0 × 10^2^	7.3 × 10^2^	8.0 × 10^2^	9.3 × 10^2^	9.0 × 10^2^

a Masses of AI region are the blood-inclusive values.

## Data Availability

The complete SAFs and S value dataset are provided via a GitHub repository available online (https://github.com/BH-Shin/JRP-respiratory_tract_SAFs_Svalues.git).
